# Major histocompatibility complex (MHC) associations with diseases in ethnic groups of the Arabian Peninsula

**DOI:** 10.1007/s00251-021-01204-x

**Published:** 2021-02-02

**Authors:** Halima Al Naqbi, Aurélie Mawart, Jawaher Alshamsi, Habiba Al Safar, Guan K. Tay

**Affiliations:** 1 Center for Biotechnology, Khalifa University of Science and Technology, Abu Dhabi, United Arab Emirates; 2 Department of Biomedical Engineering, Khalifa University of Science and Technology, Abu Dhabi, United Arab Emirates; 3 College of Medicine and Health Sciences, Khalifa University of Science and Technology, Abu Dhabi, United Arab Emirates; 4 Division of Psychiatry, Faculty of Health and Medical Sciences, The University of Western Australia, Crawley, WA Australia; 5 School of Medical and Health Sciences, Edith Cowan University, Joondalup, WA Australia

**Keywords:** MHC, HLA, Arab, Disease association

## Abstract

Since the discovery of human leukocyte antigens (HLAs), the function of major histocompatibility complex (MHC) gene families in a wide range of diseases have been the subject of research for decades. In particular, the associations of autoimmune disorders to allelic variants and candidate genes encoding the MHC are well documented. However, despite decades of research, the knowledge of MHC associations with human disease susceptibility have been predominantly studied in European origin, with limited understanding in different populations and ethnic groups. This is particularly evident in countries and ethnic populations of the Arabian Peninsula. Human MHC haplotypes, and its association with diseases, of the variable ethnic groups of this region are poorly studied. This review compiled published manuscripts that have reported a list of autoimmune diseases (insulin-dependent diabetes mellitus, systemic lupus erythematosus, myasthenia gravis, rheumatoid arthritis, psoriasis vulgaris, and multiple sclerosis) associated with MHC class I and class II in the populations of the Arabian Peninsula, specifically Bahrain, Kuwait, Oman, Qatar, Saudi Arabia, the United Arab Emirates, and Yemen. Data available was compared with other three ethnic groups, namely Caucasians, Asians, and Africans. The limited data available in the public domain on the association between MHC gene and autoimmune diseases highlight the challenges in the Middle Eastern region.

## Introduction

In the 1950s, work described by Jean Dausset, Jon van Rood, and Rose Payne laid the foundation for what is now known as the human major histocompatibility complex (MHC). The MHC region is one of the most gene-dense regions of the genome that spans 3.6 megabase contiguous sequence (The MHC sequencing consortium 1999) to an expanded 7.6 megabases (Mungall et al. 2003), on chromosome 6p21.3. Members of these MHC gene families are involved in self recognition (human leukocyte antigens (HLAs)), inflammation (tumor necrosis factor (TNF)), and components of the complement cascade (C4, C2 and complement factor B or Bf). Human MHC haplotypes may be compared among individuals and populations in the form of single nucleotide polymorphisms (SNPs), insertion-deletions (indel), as well as gross large-scale differences involving gene copy number differences. An example of these three categories of genetic variation is evident in the complement C4 gene, located in the central region of the MHC. Despite sharing 99% sequence homology, the two forms of this gene, C4A and C4B, are defined by 5 important nucleotide differences in exon 26, which contribute to isotype-specific motifs (Yu 1991; Yu et al. 1986). A HERV-K(C4) insertion in intron 9 of some, but not all, C4 genes gives rise to a long (21 kb C4L) and a short (14.6 kb C4S) form (Dangel et al. 1994; Yu et al. 1986). The complement C4 genes are segmentally duplicated as part of one to four modular RCCX cassettes, such that two to eight copies of the C4 gene may be present in a diploid genome, where each chromosome 6 consisting of one to four copies of a single C4 gene (Fernando et al. 2010).

HLA gene variants were first associated with human diseases in the late 1960s, with the discovery of the susceptibility effects of HLA-B in Hodgkin’s lymphoma (Amiel 1967). Subsequently, many other associations have been established, such as the association between HLA-A2 and acute lymphocytic leukemia (Walford et al. 1970), the classical HLA-B27 association with ankylosing spondylitis (Brewerton et al. 1973; Schlosstein et al. 1973), among others. Since then, associations with other non-HLA-genes have been implicated. For example, the presence of C4 null alleles has been associated with systemic lupus erythematosus (SLE) (Christiansen et al. 1983; Fielder et al. 1983), systemic sclerosis (Briggs et al. 1993), and myasthenia gravis (Franciotta et al. 2001). By comparing conserved extended or ancestral haplotypes of the MHC, some have argued that this C4 null association accounts for only part of the association, and have implicated the possible role of other MHC genes (Christiansen et al. 1991). Over time, the MHC has been recognized as the region of the genome with arguably the greatest number of human disease associations (Trowsdale and Knight 2013). Interestingly, the influence of MHC genes is not merely confined to immune conditions, but also includes susceptibility to neuropathologies, malignancies, and infectious diseases (Howell 2014; Trowsdale 2011). With recent advances in high-throughput DNA-based technologies and increased knowledge of the complexity and extreme polymorphism of the MHC, HLA disease associations have subsequently been reported.

## Population Definition for this Review: the Arabian Peninsula

This review focuses on the populations of a region in the Middle East, specifically, the Arabian Peninsula. The land mass of the Arabian Peninsula comprises of seven countries: Bahrain, Kuwait, Oman, Qatar, Saudi Arabia, the United Arab Emirates (UAE), and Yemen. The peninsula is surrounded by the Red and Arabian Seas, as well as the Gulfs of Arden, Oman, and Persia. It has land borders with Egypt to the west, Jordan to the northwest, and Iraq to the northeast. These connect the peninsula to Africa and the northern Levantine region, which leads to Europe and Asia. The population of the Arabian Peninsula, which is often referred to as the population of Arabian Gulf, consists of diverse ethnic groups, with influences from neighboring regions. The first inhabitants were the Bedouins, a group that dates back to at least 1200 BC. This group travelled widely throughout the Arabian Peninsula in search of water and grazing pastures, and their caravans were used by travelers from surrounding regions to traverse through the region. Through trade, populations from North and sub-Saharan Africa, the Levant, and Southern or Central Asia intermingled with the locals, with some remaining within the gulf region. A genetic study of the present-day population revealed that genetic variants of ethnic groups can be traced to Central Asia (Al-Jenaidi et al.), Southern Asia (Baluchistan), and North Africa (Iberia and Egypt) (Hajjej et al. 2018; Tay et al. 2020).

**Fig. 1:**
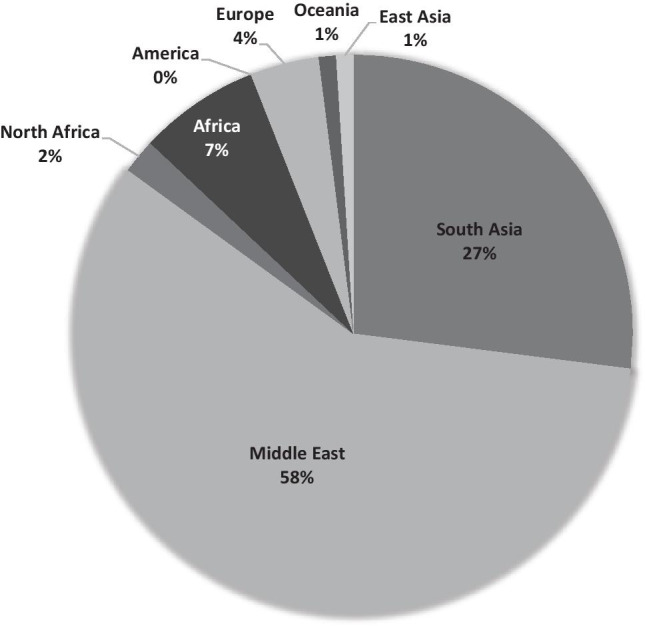
Proportions of ancestral genomes that have accumulated in the contemporary population of the UAE. Data was obtained from the genome wide analysis study of 1000 UAE citizens which provide insights into the admixture of the contemporary population. The main ancestral contribution can be traced back to South Asia (27%) which can be explained by a history of trade and exchange between the regions (Tay et al. 2020)

**Fig. 2:**
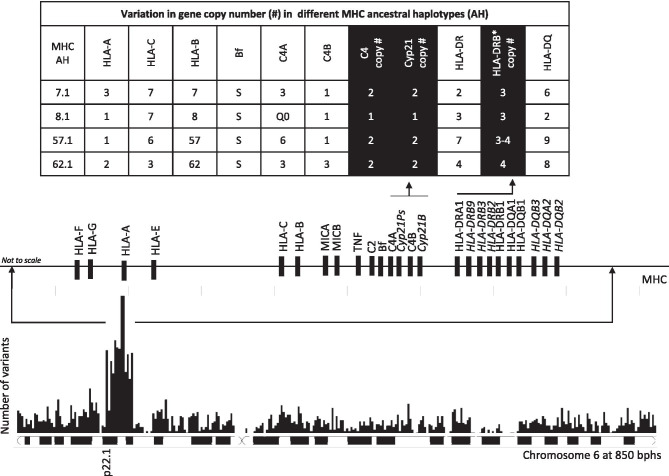
Histogram showing the distribution of variants number at chromosome 6 with troughs (low variability) and peaks (high variability) (Tay et al. 2020). Data were obtained from a genome wide association studies on 1,000 UAE citizens. Unsurprisingly, there is a significant peak in the number of variants on the short arm of chromosome 6, specifically around the MHC region. The MHC contains gene families with variable copy numbers. The size of MHC haplotypes can vary according to the gene copy number around the HLA class II and C4/CYP21 regions. The table shows the different gene copy numbers in different MHC ancestral haplotypes (Zhang et al. 1990)

A genome-wide association analyses, based on systematically dispersed SNPs across the genomes of 1000 citizens of the UAE, has provided some insight into the admixture that has occurred over history in the south eastern corner of this gulf region, with genetic influences from the Central, Southern, and sub-Saharan Africa (Fig. 1) (Tay et al. 2020). Trade and exchange throughout history has been well documented between these three regions, and as expected, there are contributions from populations from North Africa, Central Asia, and Europe through migration of people overland. A secondary route from central Africa across the Bab al-Mandab Strait has also influenced the composition of the population in this region. There has been minimal to no influence from the East Asia, Oceania, and the Americas. Although a generalization, it is not unexpected that the unique composition of the seven countries of the Arabian Peninsula has arisen from the gradual admixture of populations that have inhabited the region through interactions with those from neighboring areas (Fig. 2).

### Motivation for this review

The genetic architecture and disease susceptibility among the Arabian Peninsula region, including its MHC, remain poorly characterized (Popejoy and Fullerton 2016). The Clinical Genome Resource Ancestry and Diversity Working Group continues to highlight the importance of developing guidance for incorporating race, ethnicity, and ancestry data for applications in clinical genomics. Nevertheless, there is substantial disparity across populations in the amount of information available on clinically relevant variants in the Genome Aggregation Database (gnomAD) (Popejoy et al. 2018). This highlights the importance of implementing programs to increase the diversity of genome sequencing and clinical genomics, and reduce uncertainty of variant interpretation, especially in those that have been poorly characterized.

### Scope of this review

In keeping with the inclusion of race, ethnicity, and ancestry, this review summarizes the association between HLA class I/II genes and autoimmune diseases in populations of the Arabian Peninsula, contrasting available data with other major ethnic groups, including the Caucasian, Asian, and African populations. This review primarily focuses on a list of major autoimmune diseases, specifically insulin-dependent diabetes mellitus, systemic lupus erythematosus, myasthenia gravis, rheumatoid arthritis, psoriasis vulgaris, and multiple sclerosis. It is well established that predispositions to autoimmunity is a result of a certain disease-specific variation at HLA and non-HLA genes, in addition to epigenetic and environmental elements, which lead to the pathophysiology of the disease and its clinical features (Ramos et al. 2015). Furthermore, the already limited data on autoimmune diseases presented challenges in finding literature on associations with non-immune-related diseases.

A comprehensive search was performed on PubMed, Medline, ScienceDirect, and Google Scholar publications from database inception until April 2020. Search terms included a combination of free-text terms or controlled vocabulary terms for variations of “Arab(ian)” or countries within the region of interest (Bahrain, Kuwait, Oman, Qatar, Saudi Arabia, the United Arab Emirates (including UAE) and Yemen) and “disease association(s)” and “MHC and/or HLA”. Studies using different HLA typing methods such as serological, cellular and molecular methods were considered. Only full text articles published in English were included.

## The MHC

### General structure

The human MHC is an important region of the human genome located on chromosome 6. The boundary of the region can be fairly arbitrary and is dependent on the genes of interest at the exterminates of the region. In 1999, the MHC sequencing consortium quoted a figure of 3.6-megabases (Mb) (The MHC sequencing consortium 1999) from the HSET loci at the centromeric end to the P5-15 pseudogene in the telomeric end. Ten years later, a region of approximately 4 Mb was referred to with the MHC, containing approximately 0.5% (> 150) of the estimated 32,000 known protein coding genes in the genome (Shiina et al. 2009). Depending on the haplotype, it is reasonable to suggest that the MHC spans a 4 megabase stretch on the short arm of chromosome 6 and contains genes that are involved in numerous immune-related process (Shiina et al. 2009), as well as transplantation (Ketheesan et al. 1999; Nakamura et al. 2019; Tay et al. 1995a, b; Zou and Stastny 2009).

The detailed organization of the human MHC published in 2009 show a region that is densely packed and highly polymorphic, with multigene families (Shiina et al. 2009). One example is the HLA class II gene cluster, where each haplotype contains one copy of the HLA-DRA gene but the number of HLA-DRB genes are variable. Specifically, 9 different HLA-DRB genes have been identified in the HLA class II region, some are expressed (HLA-DRB1, HLA-DRB3, HLA-DRB4, and HLA-DRB5) and others are pseudogenes (Shiina et al. 2009). Not all HLA-DRB genes are found on a haplotype. Within the organizational structure around the C4/CYP21 region, a module known as RCCX, containing sequences of CYP21, TNX, and C4 is duplicated. However, a haplotype can contain up to four RCCX modules (Fernando et al. 2010; Traherne 2008).

These multigene families are involved in an array of different functions. Some are involved in signaling, whereas others are transcription factors of the inflammatory response. Other loci in this region encode for proteins involved in antigen processing and presentation during the adaptive immune response. Further, the region carries genes that encode for components that interact with Natural Killer (NK) cells and cytokines, as well as components of the complement cascade (Shiina et al. 2009).

### Genes of the MHC

Genes across the MHC region encode proteins that are important for self-recognition (e.g., HLA), inflammation (e.g., TNF) and the complement cascade (e.g., C4, C2, and Bf). Many of these genes tend to be highly polymorphic, some of which are duplicated and present in multiple copies (Dawkins et al. 1999).

The highly polymorphic classical class I genes (HLA-A, HLA-B, HLA-C) encode peptide binding receptors on the cell surface of all nucleated cells. The class I heterodimeric cell surface receptor platform contains two polypeptide heavy α chains and a β-2 microglobulin, where a peptide-binding groove is located between the α1 and α2 domains. These receptors are responsible for presenting peptide fragments of non-self-antigens to cytotoxic CD8+ T cell receptors (TCRs). The activation of the T cells could lead to tolerance, if the presented peptides are identified as “self,” initiation of cell-mediated immunity in case of “non-self” antigens, or to a maladaptive autoimmune response in the presented “self” peptide was misidentified.

On the other hand, the non-classical class I genes such as HLA-F, HLA-G, and HLA-E are characterized by limited levels of polymorphisms and lower cell surface expression compared to the classical class I genes (Shiina et al. 2009). Interspersed between the classical HLA class I genes are loci that encode for the MHC class I chain related (MIC) proteins, MICA and MICB (Bahram et al. 1994; Leelayuwat et al. 1994). These proteins do not appear to have a role in antigen presentation but act as a stress-induced self-antigen that is recognized by γδ T cells, natural killer (NK), and CD8+ T cells (Ghadially et al. 2017).

The classical HLA class II multigene family (HLA-DR, HLA-DQ, and HLA-DP) are expressed on the surface of antigen-presenting cells (e.g., dendritic cells, macrophages, and B cells), involved in presenting peptides to CD4+ T cells (Wieczorek et al. 2017). The HLA class II molecules include two polypeptide chains, an α and a β chain (Alfonso and Karlsson 2000). There are a number of non-classical HLA class II loci, which include HLA-DM and HLA-DO. HLA-DM is involved in peptide loading of class II molecules in the endosomal/lysosomal system, whereas HLA-DO acts as a chaperon for antigenic peptides (Alfonso and Karlsson 2000).

Flanked by the HLA class I and HLA class II regions, the central MHC also carries a number of multigene families including complement factor genes (C2, C4, and CFB), cytokine genes (TNF, LTA and LTB) (Shiina et al. 2009), and other genes that are not linked to the immune function or inflammation. Many of these genes are duplicated and polymorphic (Zhang et al. 1990). The TNF is a multifunctional cytokine, secreted by macrophages and T lymphocytes, involved in systematic inflammation. It regulates immune cells and protects against tumors. The three complement genes (C2, C4, and Bf) in the central portion of the MHC encode for proteins involved in the early stages of the complement cascade. As mentioned previously, the actual number of C4 genes can vary from one to four per haplotype or two to eight per diploid genome (Bánlaki et al. 2012; Castley and Martinez 2012; Fernando et al. 2008) and it is dependent on the ancestral haplotype (AH) (Zhang et al. 1990). In this regard, Zhang et al. (1990) has suggested that relative deficiencies of the products of the central MHC genes could impact the susceptibility to autoimmune diseases (Zhang et al. 1990).

The extensive degree of polymorphism of the MHC genes found in humans at the population level is thought to contribute to the success of displaying a broad range of non-self peptides to the T cell antigen receptor. These variants are partly caused by point mutations or gene conversion. Variation in HLA genes that produce amino acid changes, including those in the peptide-binding groove of the MHC molecules, can alter the binding preferences, leading to a different peptide-binding repertoire (Dendrou et al. 2018). If a change in the HLA molecule provides fitness advantage to its host, it will be maintained in the population. Hence, it is not surprising that many HLA genes are associated with hundreds of diseases, owing to their polymorphic nature and role in immune response initiation (van Deutekom and Keşmir 2015).

Interestingly, an HLA molecule could increase the likelihood of developing a particular disease, while a closely related HLA molecule cannot. For instance, HLA-B*27:05 has been found to cause ankylosing spondylitis, while HLA-B*27:09 does not have an effect in the disease predisposition (Fiorillo et al. 1998). Even though the difference between both alleles is minimal, and mainly at the peptide-binding groove, it produces an entirely different outcome (van Deutekom and Keşmir 2015). HLA-B is known to have the highest number of alleles, but HLA-DR/DQ subregion has been associated with the highest number of diseases (Kishore and Petrek 2018).

The reporting and naming of HLA alleles and haplotypes in this review follows the HLA nomenclature system described by Marsh (Marsh et al. 2010). The asterisk “*” sign indicates that the typing has been performed by a molecular method. The digits before the first colon (field 1) define the type or the allele group. The next set of digits (field 2) denotes the subtype, and the third set of digits is used to show synonymous variants. Many studies reported in this review describe HLA allelic associations to 2-digit (first field) or 4-digit (second field) resolution.

### HLA allele diversity and MHC haplotypes

According to the international HLA nomenclature committee, there were 19,031 and 7183 HLA class I and class II alleles, respectively, in December 2019. The most frequent haplotypes observed were the AH 8.1 haplotype (*HLA-A1-B8-DR3*) in Caucasians; the HLA-A*33:03-B*58:01-DRB1*03:01 combination in Asians, and the HLA-A*30:01-B*42:01-DRB1*03:02 haplotype in Africans (Gourraud et al. 2014). To date, no large-scale study has characterized ancestral haplotypes in the ethnic populations of Arabs. Based on recent studies of HLA frequencies in the population of the UAE, the most frequent HLA-A/HLA-B combinations are HLA-A*02-B*50, HLA-A*02-B*51, HLA-A*26-B*08, HLA-A*11-B*40, and HLA-A*03-B*50 (Kulski et al. 2019). Only two haplotypes from the list of the most frequent haplotypes observed in Caucasians, Asians, and Africans from the 1000-genome project were observed in the top 10 HLA-A-B haplotypes in UAE. In Saudi Arabia, HLA-A*02, HLA-B*50, and HLA-B*51 were the most frequent HLA alleles described in the population; whereas in neighboring Oman, HLA-A*02, HLA-B*08, and HLA-B*50 were the most frequent alleles (Hajeer et al. 2009).

## Disease associations and the Arabian MHC

### Insulin-dependent diabetes mellitus

Insulin-dependent diabetes mellitus (IDDM), also known as type 1 diabetes (T1D), is an autoimmune disease where pancreatic islet β cells are destroyed by the immune system, resulting in low production of insulin and insulin deficiency. Consequently, the control of blood sugar level is disrupted, leading to complications that causes serious health issues and shortened life expectancy (Polychronakos and Li 2011).

IDDM can affect individuals at any age, but usually develops at a young age making it a common childhood disease. The incidence of the IDDM among children and adolescents in Saudi Arabia, Algeria, and Morocco rank in top 10 countries worldwide. The Middle East and North African (MENA) region ranks third region in terms of number of children and adolescents with IDDM (Nam Han et al. 2017). Epidemiological studies in Arab countries show that there are between 31.4 and 37.1 new cases per 100,000 population (Al-Herbish et al. 2008).

Genome-wide association studies (GWAS) have highlighted the complexity of IDDM and have implicated the involvement of numerous genes in the development of the disease (Noble and Valdes 2011). The insulin (INS), inositol 1-, 4-, 5-trisphosphate receptor type 3 (ITPR3), cytotoxic T-lymphocyte-associated protein 4 (CTLA4), protein tyrosine phosphatase, non-receptor type 22 (PTPN22), and interleukin 2 receptor alpha (IL2RA) genes are non-HLA risk loci that have been confirmed (Steck and Rewers 2011).

The HLA genes represent almost half of the genes found to be associated with IDDM. The association of the disease with HLA class II (HLA-DR, HLA-DQ, and HLA-DP) have been found to be linked to the geographic location or racial background of individuals (Table 1) (Ikegami et al. 2006). Alleles that have a strong predisposition to the disease include HLA-DPB1*04:02, HLA-DPB1*03:01, and HLA-DPB1*02:02 (Steck and Rewers 2011). The strong linkage disequilibrium between the genes within the MHC region makes it difficult to evaluate the direct effect of each gene separately. Interestingly, studies on Caucasian families showed that a number of HLA class I alleles that contributes to the onset of IDDM are also associated with type 2 diabetes (T2D) (Jahromi and Al-Ozairi 2019).

**Table 1:** IDDM and associated susceptible and protective HLA alleles and haplotypes in different populations. Alleles or haplotypes in brackets are protective

	Insulin-dependent diabetes mellitus							
Ethnicity	Alleles							Reference
	HLA-A*	HLA-C*	HLA-B*	HLA-DRB1*	HLA-DQA1*	HLA-DQB1*	HLA-DPB1*	
Caucasian	30	03, 08, 16	18, 19, 44, 39	04:02, [04:03], 04:04, 04:05		[03:01], 03:02	02:02, 03:01, [04:02], [04:03]	(Steck and Rewers 2011)
		03:03, 08:02, 16:01, 07:01, 07:02	18:01, 39:06, [44:03], [38:01]					(Varade et al. 2018)
		02:01, 11:01, 24:02, 3201, 6601	07:02, 18:01, 35:02, 39:06, 44:03, [57:01]				[01:01], [02:02], [04:01], [04:02], [17:01]	(Noble and Valdes 2011)
Asian	24:02, 24:03, [11:01]	01:02, 03:02, 15:02, 07:02	[13:01], 58:01	04:05		[01:01], [05:03]	02:02	(Bugawan et al. 2002)
African				03:01, 04, 09	03	02, 03:02		(Pirie et al. 2001)
Saudi Arabian				[04:01]				(Al-Hussein et al. 2003)
Bahraini				03:01:01, 04:01:01, [10:01:01], [11:01]		02:01, [03:01:01], 03:02, [05:01:01]		(Al-Harbi et al. 2004)

	Haplotypes							
Caucasian	DRB1*03:01-DQA1*05:01- DQB1*02:01							(Erlich et al. 2008)
	DRB1*04:05-DQA1*03:01-DQB1*03:02							
	DRB1*04:02-DQA1*03:01-DQB1*03:02							
	DRB1*04:01-DQA1*03:01-DQB*03:02							
	DRB1*04:04-DQA1*03:01-DQB1*03:02							
	DRB1*08:01-DQB1*04:01-DQB1*04:02							
	[DRB1*15:01-DQA1*01:02-DQB1*06:02]							
	[DRB1*14:01-DQA1*01:01-DQB1*05:03]							
	[DRB1*07:01-DQA1*02:01-DQB1*03:03]							
	DRB1*04:01/02/04/05/08-DQA1*03:01-DQB1*03:02/04							(Noble and Valdes 2011)
Asian	DRB1^∗^04:05-DQB1^∗^04:01							(Ikegami et al. 2007)
	DRB1^∗^09:01-DQB1^∗^03:03							
	DRB1*04:05-DQB1*04:02							
	[DRB1*15:02-DQB1*05:01]							(Bugawan et al. 2002)
	[DRB1*08:03-DQB1*06:01]							
	[DRB1^∗^15:01-DQB1^∗^06:02]							
African	DRB1*03:01-DQA*05:01							(Pirie et al. 2001)
	DRB1*04-DQA*03							
	DRB1*04-DQB*03:02							
	DRB1*03:01-DQB*02:01							
	DQA*05:01-DQB*02:01							
	DQA*03-DQB*03:02							
Bahraini	DRB1*03:01:01-DQB1*02:01							(Al-Harbi et al. 2004)
	DRB1*03:01:01-DQB1*02:01							
	DRB1*03:01:01-DQB1*03:02							
	[DRB1*10:01:01-DQB1*05:01:01]							
	[DRB1*11:01:01-DQB1*03:01:01							
	[DRB1*11:01:01-DQB1*05:01:01]							
Bahraini	DRB1*03:01:01-DQB1*02:01							(Al-Jenaidi et al. 2005)
	DRB1*04:01:01-DQB1*02:01							
	[DRB1*07:01:01-DQB1*02:01]							
	[DRB1*11:01:01-DQB1*03:01:01]							
	DQA1*03:01-DQB1*02:01							(Zayed 2016)
	DQB1*02:01-DRB1*03:01							
	DRB1*04:05-DQB1*03:02							
Saudi Arabian	DRB1*04-DQB1*02:01/02:02							(Al-Hussein et al. 2003)

The increasing incidence of IDDM among the populations of the Arabian Peninsula is thought to be due unhealthy lifestyle choices, changes in breastfeeding practices, higher hygiene standards, and factors that contribute to vitamin D deficiency. Moreover, the high rates of endogamous and consanguineous marriages are also believed to have contributed to the higher prevalence of the IDDM and other autoimmune diseases (Jahromi and Al-Ozairi 2019).

A study in Saudi Arabia that was conducted on IDDM patients revealed a significant correlation between the disease and the haplotypes HLA-DRB1*04-DQB1*02:01/02:02. The same haplotype association, known as *HLA-DR4*, was also found in Caucasian (Noble and Valdes 2011) and Japanese patients (Ikegami et al. 2007). Further, HLA-DPB1*04:01 was found to be significantly protective in the Saudi Arabian population (Al-Hussein et al. 2003).

Studies on the population of Bahrain reported a high frequency (70.1%) of haplotypes HLA-DRB1*03:01:01-DQB1*02:01 and HLA-DRB1*03:01:01-DQB1*03:02 in IDDM patients (Al-Jenaidi et al. 2005). The HLA-DRB1*03:01:01-DQB1*02:01 haplotype (also known as *HLA-DR3*) was also found to have a great susceptibility to the disease in Caucasians (Noble and Valdes 2011). In contrast, the MHC haplotypes HLA-DRB1*10:01:01-DQB1*05:01:01, HLA-DRB1*11:01:01-DQB1*03:01:01, as well as HLA-DRB1*11:01:01-DQB1*05:01:01 were found to be protective in the patients from Bahrain (Table 1) (Al-Harbi et al. 2004; Al-Jenaidi et al. 2005).

### Systemic lupus erythematosus

Systemic lupus erythematosus (SLE) is a chronic multisystem autoimmune disease that exhibits variable phenotypes and severity depending on ethnicity (Adwan 2018). Women are predominantly affected (Feldman et al. 2013). Clinical features include hematological abnormalities, skin, joint diseases, renal disease, and neuropsychiatric complications (Feldman et al. 2013). Although clinical expression studies of SLE in Arabian populations are limited, similar phenotypic features and disease factors have generally been observed when compared to other populations (Adwan 2018).

The prevalence of SLE among populations of the Arabian Gulf region has been shown to be higher than most other ethnicities. Specifically, a recent epidemiological study of the population of the UAE revealed an age standardized rate of 103 per 100,000 population, which is higher than most Caucasian populations (Al Dhanhani et al. 2017).

SLE is a multifactorial disease where genetics, hormonal, and environmental factors are involved (Hachicha et al. 2018). GWAS studies have highlighted over 80 loci with variants that contribute in a cumulative way (Morris et al. 2012; Yeoh et al. 2018) The exact weight of each genetic factor has yet to be determined, but genes of the MHC region seem to be heavily involved. HLA class II genes, particularly HLA-DRB1, HLA-DQB1, and HLA-DQA1, have been reported as the strongest risk factors, and some alleles have been associated with particular clinical phenotypes (Graham et al. 2007; Wadi et al. 2014).

Some of the alleles are not directly linked to the SLE disease but associated to clinical phenotypes of SLE. For example, HLA-DQB1*03 seems to be related with skin manifestations, HLA-DRB1*15 with nephritis, DRB1*10 with hematological manifestations, and HLA-DRB1*11 with neurological manifestations (Ke et al. 2018; Wadi et al. 2014).

A worldwide recurrent association has been observed for MHC class II alleles HLA-DRB1*03:01, HLA-DRB1*15:01, and HLA-DQB1*06:01 (Table 2) (Al-Motwee et al. 2013; Fernando et al. 2008). Additionally, a protective effect has been reported for HLA-DRB1*14:01 (Graham et al. 2007).

**Table 2:** SLE and associated susceptible and protective HLA alleles and haplotypes in different populations. Alleles or haplotypes in brackets are protective

	Systemic lupus erythematosus							
Ethnicity	Alleles							Reference
	HLA-A*	HLA-C*	HLA-B*	HLA-DRB1*	HLA-DQA1*	HLA-DQB1*	HLA-DPB1*	
Caucasian				03:01, 15:01	04:01, 04:02			(Fernando et al. 2008)
				03:01	05:01, 01:02	02:01, 06:02		(Sirikong et al. 2002)
				[07], 03				(Hachicha et al. 2018)
				[13], 15:01				(Bettencourt et al. 2015)
Asian				15:01, 08:02, 09:01	[01:02]			(Hachicha et al. 2018)
				15:02,15:01,16:02, [12:02]		15:01, [03:01]		(Sirikong et al. 2002)
African				03, 01				(Hachicha et al. 2018)
Saudi Arabian	29		51	[04], 15, [16]		06		(Al-Motwee et al. 2013)
Saudi Arabian	29		51	10, 11, 15, [16]				(Wadi et al. 2014)

	Haplotypes							
Caucasian	DRB1*15:01/DQB1*06:02							(Fernando et al. 2008)
	DRB1*03:01-DQB1*02:01							
	DRB1*08:01-DQB1*04:02							
	DRB1*15:01-DQB1*06:02							(Graham et al. 2007)
	DRB1*08:01-DQB1*04:02							
Asian	DRB1*15:01-DQB1*06:02							(Sirikong et al. 2002)
	DRB1*15:02-DQB1*05:01							
	[DRB1*12:02-DQB1*03:01]							
Saudi Arabian	DRB1*15-DQB1*06							(Al-Motwee et al. 2013)

Other MHC genes have also been associated to SLE, including genes of the complement cascade (C4A and C4B) and TNF. However, LD between these genes and some HLA haplotypes make the interpretation of their role in SLE difficult (Fernando et al. 2008).

HLA-DRB1*15-DQB1*06 has been confirmed as a risk haplotype in Saudi Arabian SLE patients (Table 2). However, the HLA-DQB1*06 allele frequency (40%) was higher than that of HLA-DRB1*15 (20%), suggesting that HLA-DQB1 might represent an independent association with the disease. Moreover, the study found that alleles HLA-DRB1*16 and HLA-DRB1*04 are protective against the disease (Table 2) (Al-Motwee et al. 2013). Other associations with SLE have also been found within MHC class I, including HLA-A*29 and HLA-B*51 (Al-Motwee et al. 2013).

Some contrasting results have been observed depending on ethnicity. For example, *HLA-DRB3* was found to be a protective allele among Saudi Arabians (Wadi et al. 2014), contradicting the studies from other populations including Malaysians (Chai et al. 2012) and Jamaicans (Smikle et al. 2002).

### Myasthenia gravis

Myasthenia gravis (MG) is a rare, chronic autoimmune neuromuscular disease that causes weakness in the skeletal muscles. The disease is a result of defects in the transmission of nerve impulses to muscles due to autoantibodies that target molecules at the neuromuscular junction. This causes impairment for the neuromuscular transmission in 80% of the patients (Avidan et al. 2014). The autoantibodies can act opposing to the acetylcholine receptor (AChR), the muscle-specific kinase (MuSK), or the lipoprotein-related protein 4 (LRP4). Moreover, the disease can be categorized based on the age of onset, thymic abnormalities, ocular involvement and type of autoantibodies (Avidan et al. 2014).

The disease can be further classified into early-onset MG (EOMG), where patients are less than 50 years old, and late-onset MG (LOMG), where patients are more than 50 years old. Both EOMG and LOMG have different HLA allele(s) associations and disease characteristics. For example, EOMG is more common in females and LOMG in males (Feng et al. 2019).

The prevalence of MG among European societies has been estimated to be around 1 to 5000 (Berrih-Aknin 2014). On the other hand, there has been no data available on the prevalence of the disease in the population of the Arabian Peninsula to date.

The relatively low prevalence of the disease has made it difficult for GWAS efforts to cover all probable heritability factors and confirm the specific genetic susceptibility factor(s). In addition, the LD between the MHC genes, including HLA, has also made it difficult to predict the exact susceptibility gene.

Other non-HLA genes have been shown to be linked to EOMC, including interleukin-10 (IL-10) and cellular tyrosine phosphatase 22 (PTPN22) on chromosome 1, cytotoxic T cell late antigen 4 (CTLA4) and muscle nicotinic acetylcholine receptor α-subunit (CHRNA-1) on chromosome 2, TNF-α on chromosome 6, and TNFAIP3-interacting protein 1 (TNIP1) on chromosome 5 (Avidan et al. 2014).

In a study by Hajeer et al. (2009) involving patients from Saudi Arabia, the haplotype comprising of HLA-B*08 and HLA-DRB1*03 was found to be frequent in MG patients. Further, HLA-B*08 showed the strongest susceptibility effect and was linked to young age at onset, and with the female gender. Similarly, a recent study performed on Swedish patients with AH8.1 showed that HLA-B*08:01 is the unique risk factor that marked EOMG (Table 3) (Varade et al. 2018).

**Table 3:** MG and associated susceptible and protective HLA alleles and haplotypes in different populations. Alleles or haplotypes in brackets are protective

	Myasthenia gravis							
Ethnicity	Alleles							Reference
	HLA-A*	HLA-C*	HLA-B*	HLA-DRB1*	HLA-DQA1*	HLA-DQB1*	HLA-DPB1*	
Caucasian				05:01, 05:02, 15:01, 16				(Avidan et al. 2014)
			08:01					(Varade et al. 2018)
Asian				09, [08]				(Xie et al. 2011)
	02:07		46:01	09:01				(Feng et al. 2019)
African	No data or research							
Saudi Arabian	01, 23		08, 18, [50], 51	[07], [10], 11, 13, 16				(Hajeer et al. 2009)

	Haplotypes							
Caucasian	A1-B8-DR3-DQ2							(Avidan et al. 2014)
Asian	A*02:07:01-C*01:02:01-B*46:01:01-DRB1*09:01:02-DQA1*01:01:01-DQB1*03:03:02							(Feng et al. 2019)
African	No data or research							
Saudi Arabian	B*08-DRB1*03							(Hajeer et al. 2009)

Interestingly, HLA-B*51 was found to be another risk allele in the population of Saudi Arabia, with an allele frequency of 19.3% in the general population (Hajjej et al. 2018). Another observation was the protective effect of HLA-B*50, a common allele in Saudi Arabians, but rarely found in other ethnicities (Hajeer et al. 2009).

### Rheumatoid arthritis

Rheumatoid arthritis (RA) is an autoimmune disease that affects the joints. This long-term disease results in swollen and painful joints, most commonly the wrist and the hands (McInnes and Schett 2017). The annual incidence of RA worldwide is around 3 cases per 10,000 (Saxena et al. 2017). However, the prevalence of RA in populations of the Arabian Peninsula remains uncertain.

Genetic factors, when combined with environmental events, can define the pathogenesis of the disease. Multiple pathways, including environmental factors (e.g., smoking, obesity, and vitamin D) and mucosal microbiome, seem to be involved, resulting in the same clinical phenotype. Low concordance has been observed between twins, suggesting a greater contribution from environmental factors (Firestein and McInnes 2017).

Despite more than 100 non-MHC candidates identified, genes within the MHC have long been regarded as the major genetic risk factor of the disease (Kunz and Ibrahim 2011). The individual contributions of non-MHC genes seem to be low, but when combined with others, have been suggested to increase the risk by interaction (Kurkó et al. 2013; Okada et al. 2014). Among these gene variants, PTPN22 (1p13.2), PAD14 (1p36.13), and CDK6 (7q21.2) are known to play an important role in increasing the risk of the disease (Snir et al. 2014).

Gene(s) within the MHC class II region are considered to be the main genetic contributor, accounting for more than 40% of the genetic risk (Firestein and McInnes 2017). The significant genetic risk of RA has been primarily explained by the shared epitope (SE) hypothesis proposed by Gregersen et al. (1987). SE refers to an amino acid sequence motif with residues 67 to 74 overrepresented by a number of HLA-DRB1 alleles in RA patients. The SE is thought to act as a “signal transduction ligand that interacts with an evolutionarily-conserved receptors” (de Almeida et al. 2011). Their shared amino acid sequences (QKRAA, QRRAA, and RRRAA) are located at positions 70 to 74 of the third hypervariable region of the HLA-DRB1 antigen-binding groove (Madeleine et al. 2010). The amino acid motif with the strongest association is QKRAA, encoded by HLA-DRB1*04:01, followed by QRRAA encoded by HLA-DRB1*04:04, HLA-DRB1*01:01 and HLA-DRB1*04:05. Finally, the rarest of the 3 SE motifs, RRRAA, is encoded by DRB1*10:01 (de Almeida et al. 2011). It has been found that more than 90% of RA patients express at least one of these SE alleles (Firestein and McInnes 2017). Other alleles that exhibit risk to RA include HLA-DRB1*01:02, HLA-DRB1*04:08, HLA-DRB1*04:10, HLA-DRB1*10:01 (Table 4). These alleles share one of the three SE homologous amino acid sequence variants that are associated with increased severity of RA in Caucasians (Madeleine et al. 2010).

**Table 4:** RA and associated susceptible and protective HLA alleles and haplotypes in different populations. Alleles or haplotypes in brackets are protective

	Rheumatoid arthritis							
Ethnicity	Alleles							Reference
	HLA-A*	HLA-C*	HLA-B*	HLA-DRB1*	HLA-DQA1*	HLA-DQB1*	HLA-DPB1*	
Caucasian				04:01, 04:04, 01:01, 04:05, 10:01				(de Almeida et al. 2011)
				01:01, 01:02, [01:03], 04:01, 04:04, 04:05, 04:08, [07], 10:01, [12:01], [13:01], 14:02, [15:01]				(Gough and Simmonds 2007)
				[01:03], [04:02], [13:01], [13:02]				(van Drongelen and Holoshitz 2017)
Asian				01:01, [03:01], 04:01, [04:03], 04:05, [04:06], 04:10, [07:01], 10:01, [13:01], [14:05]				(Jun et al. 2007)
African	No data or research							
Saudi Arabian				04:05, [06], 08, 10				(Al-Swailem et al. 2006)
Kuwaiti				03:07, 03:08, 11, 15				(Alsaeid et al. 2002)

	Haplotypes							
Caucasian	DRB1*04:01-DQA1*03-DQB1*03:01							(van Drongelen and Holoshitz 2017)
	DRB1*04:01-DQA1*03-DQB1*03:02							
	DRB1*04:04-DQA1*03-DQB1*03:02							
Arabian	No data or research							

The role of these SEs is not fully understood but recent studies have highlighted an increase of efficiency in peptide presentation to T cells for altered citrullinated peptides. This results in production of interleukin 17 (IL-17), interferon gamma (IFN-γ), and anti-citrullinated peptide antibodies (ACPA), which are seen in 80 to 90% of the patients (Firestein and McInnes 2017).

Furthermore, a list of diseases has also been associated to the same SEs including polymyalgia rheumatica, giant cell arteries, IDDM, SLE, autoimmune hepatitis, chronic lymphoid leukemia, and psoriatic arthritis (de Almeida et al. 2011).

A study conducted on RA patients in Saudi Arabia using a polymerase chain reaction sequence specific primers (PCR-SSP) method revealed that the strongest risk allele is HLA-DRB1*04 followed by HLA-DRB1*08 and HLA-DRB1*10, when compared with healthy controls (Al-Swailem et al. 2006). Further subtyping of the HLA-DRB1*04 locus of the patients has showed strong association with 11 subtypes, specifically HLA-DRB1*04:01, HLA-DRB1*04:02, HLA-DRB1*04:03:01, HLA-DRB1*04:03:02, HLA-DRB1*04:04, HLA-DRB1*04:05, HLA-DRB1*04:06, HLA-DRB1*04:08, HLA-DRB1*04:10, HLA-DRB1*04:14, and HLA-DRB1*04:24.

Further comparison has indicated a significantly positive association of the disease to HLA-DRB1*04:05, which is one of the shared epitopes. A negative association of HLA-DRB1*04:03 with RA has also been reported (Table 4) (Al-Swailem et al. 2006). Surprisingly, a significant protective effect of RA has been observed in HLA-DRB1*06 indicating that HLA-DRB1 could have both negative and postive correlation with the disease (Al-Swailem et al. 2006).

A study on 69 children in Kuwait with juvenile Rheumatoid arthritis using PCR-SSP showed a high incidence of *HLA-DR3* in patients, when compared to the control group. The study revealed that this was due to the significant contribution of HLA-DRB1*03:07 and HLA-DRB1*03:08 alleles (Table 4) with no other HLA-DR alleles found in this study (Alsaeid et al. 2002).

A recent pan-Arabian RA genetics GWAS study of patients from the UAE, Saudi Arabia, Qatar, Lebanon, and Jordan reported replication of associated HLA alleles to those of European ancestry. Imputation of the HLA alleles and the HLA amino acid polymorphisims have confirmed the significant effect of HLA-DRB1 amino acid position 11 to RA predisposition in Arabs. Moreover, two novel Arab-specific loci reached genome-wide significance in association analyses of RA (Saxena et al. 2017).

### Psoriasis vulgaris

Psoriasis vulgaris (PV) is a common hyper proliferative dermatosis that causes the skin to flake particularly around joints. It is characterized by abnormal symmetrical epidermal proliferation accompanied by an inflammatory response. The disease affects around 1% of the world population, but its prevalence in Arabian populations has not been accurately defined (Capon 2017). Potential triggers of PV are believed to involve a combination of genetic and environmental factors including physical trauma, infections, stress, alcohol, smoking, and obesity (Capon 2017).

Genetic studies have revealed more than 30 SNPs associated with PV, affecting both the skin and the immune system. Nevertheless, only two SNPs have been found to cause the disease independently. GWAS have revealed that the MHC region accounts for the highest association signal observed in patients with threshold of *P* < 5.0 × 10^–8^ (Ayala-Fontanez et al. 2016). The most significantly associated alleles, being HLA-C*06 and HLA-B*57, have been described by Schmitt-Egenolf et al. (1993). The alleles appear to mark MHC haplotypes with the greatest disease risk. In particular, the association with HLA-C*06 allele is very strong and has been found in more than 60% of psoriasis patients (Table 5) (Ayala-Fontanez et al. 2016). Moreover, the ancestral haplotype (AH), specifically AH57.1 (HLA-C*06-B*57-DRB1*07:01-DQA1*02:01-DQB1*03:03), was the first to be observed in PV with 26 times higher frequency in patients than in controls (Szczerkowska-Dobosz 2005). Further, differential expression of the MICA and MICB in the class I region of the MHC have been found to be potentially implicated in the etiology of the disease (Tay et al. 2000).

**Table 5:** PV and associated susceptible and protective HLA alleles and haplotypes in different populations. Alleles or haplotypes in brackets are protective

	Psoriasis vulgaris									
Ethnicity	Alleles									Reference
	HLA-A	HLA-C*	HLA-B	HLA-DRB1*		HLA-DQA1*		HLA-DQB1*	HLA-DPB1*	
Caucasian		06		07:01		02:01		03:03		(Szczerkowska − Dobosz 2005)
		06:02, 07:01, 07:02, 07:04, 12:02	*27, 57							(Prinz 2018)
Asian	*02:07	06:02, 07:04, 12:02								(Prinz 2018)
	*26	06:02, [03:04]	*13, 27							(Zhang et al. 2003)
African	No data or research									
Omani	[68]		52							(Al-Mamari et al. 2009)

	Haplotypes									
Caucasian	C*06-B*57-DRB1*07:01-DQA1*02:01-DQB1*03:03								(Szczerkowska − Dobosz 2005)	
Asian	A*26-C*06:02-B*27								(Zhang et al. 2003)	
African	A*01-B*57-DRB1*07:01-DQA1*02:01-DQB1*03:03								(Szczerkowska − Dobosz 2005)	
Arabian	No data or research									

A study in Oman conducted on 55 local PV patients showed contradicting disease association results. The *HLA-C6* antigen was reported in 27.8% of the patients and 16% in controls, but showed no significant contribution to disease risk (Al-Mamari et al. 2009). Moreover, *HLA-B57* was reduced in both groups and no increase was observed for *HLA-DR7* in patients. A novel association, with *HLA-B52*, was reported as a potential risk allele for PV in the Arabian population of Oman. The study also reported a negative association between a number of HLA alleles and the disease including *HLA-A68*, *HLA-DR13*, *HLA-DR52*, and *HLA-DQ2* (Table 5) (Al-Mamari et al. 2009). Although the result of this study suggests that there are selective racial and environmental factors contributing to the etiology of the disease the sample size is too small and larger regional efforts will be required.

### Multiple sclerosis

Multiple sclerosis (MS) is demyelinating disease of the central nervous system where the immune system attacks the myelin sheath of the neurons. This causes disruptions in sensory, motor, autonomic and neurocognitive functions. The incidence of the disease is approximately tenfold higher risk in the Caucasian population, when compared to other populations from around the world, with a prevalence of approximately 108 per 100,000. Women and young adults with ancestry from Northern and Central Europe were found to be particularly at a higher risk of developing MS (Mohammed 2016). There has been an increase in the number of MS diagnosed in the Arabian Gulf region, rising from 31 to 55 MS cases per 100,000 individuals in a 2016 report (Mohammed 2016).

Around 50 genome-wide screens have been performed to investigate the role of MHC and non-MHC genes in the risk to MS. However, as suggested by Schmidt et al. (2007), MS is not a homogenous disease with a single-locus model, but a collection of diseases with different genetic etiologies (Schmidt et al. 2007).

Possible non-MHC genetic risk of the disease includes the myelin oligonucleotide glycoprotein (MOG), which lies in the MHC class I region (Wadi et al. 2014). It represents a component of the myelin expressed on the outer surface of the sheath that wraps around the neurons and exons. It is believed that when disrupted, it could influence the action potential electrical conduction that might lead to MS. However, its function in the progression of the disease has been questioned in recent years (Bronge et al. 2019).

Case–control and family-based research have revealed that haplotype DRB1*15:01-DQA1*01:02-DQB1*06:02 is strongly associated to MS in different ethnic groups (Schmidt et al. 2007). Interestingly, this haplotype has also been linked to other diseases, including narcolepsy and SLE (Mohammed 2016). In Saudi Arabian patients, HLA-DRB1*15:01, HLA-DQB1*06:02, and HLA-DQB1*06:01 have been shown to confer the highest risk to MS, with HLA-DRB1*15:01 showing an increased risk of previous relapses. The HLA-DRB1*15:01 allele seems to present the largest risk to MS in populations around the world including Caucasians (Patsopoulos et al. 2013), the Chinese (Qiu et al. 2011), and some populations in the Middle East (Galehdari et al. 2018) (Table 6).

**Table 6:** MS and associated susceptible and protective HLA alleles and haplotypes in different populations. Alleles or haplotypes in brackets are protective

	Multiple sclerosis							
Ethnicity	Alleles							Reference
	HLA-A	HLA-C*	HLA-B	HLA-DRB1*	HLA-DQA1*	HLA-DQB1*	HLA-DPB1*	
Caucasian	[*02:01]		*37:01	03:01, 04:04, 13:03, 14:01, 15:01		06:02		(Patsopoulos et al. 2013)
Asian				[09], 15				(Qiu et al. 2011)
African	No data or research							
Saudi Arabian				15:01		02:01, [02:02], [05:03], 06:02, 06:03, [06:14]		(Al Jumah et al. 2018)
	2, 9, 19		5, 35, 40					

	Haplotypes							
Caucasian	DRB1*15:01-DQA1*01:02-DQB1*06:02							(Patsopoulos et al. 2013)
Asian	DRB1*16:02-DPB1*05:01							(Qiu et al. 2011)
Saudi Arabian	DRB1*15:01-DQB1*06:02							(Al Jumah et al. 2018)
	DRB1*03:01-DQB1*02:01							
Bahraini	A*02-B*40-DR2							(Al-Nashmi et al. 2018)
Kuwaiti	A*02-B*40-DR2							(Al-Shammri et al. 2004)

On the other hand, the HLA-DQB1*02:03, HLA-DQB1*05:03, and HLA-DQB1*06:14 alleles were not found in the Saudi Arabian MS group (Table 6) (Al Jumah et al. 2018). Further, the same study showed that patients that carried HLA-DRB1*15:01 and HLA-DQB1*06:02 had lower levels of vitamin D in serum when compared to those without these alleles (Al Jumah et al. 2018). The vitamin D effect is of a particular interest as it has been linked to MS susceptibility and the alteration of MHC class II gene expression (Handunnetthi et al. 2010). Specifically, a previous study by Ramagopalan et al. (2009) has revealed that the expression of HLA-DRB1*15:01 may be controlled by vitamin D. More research is required to investigate the interaction between HLA allele expression and vitamin D, especially in the populations of the Arabian Gulf where vitamin D deficiency is common (Singh et al. 2019).

A study conducted on MS patients in Bahrain using tissue typing methods by polymerase chain reaction (PCR) reported a number of HLA associations, suggesting that there might be population-specific genetic factors that contribute to susceptibility or protection to MS among the populations of the Arabian Gulf. The study did not find any significance between HLA-DRB1*15:01 allele and patients from Bahrain. A number of HLA class I alleles, specifically *HLA-A19*,* HLA-A2*,* HLA-A9*,* HLA-B35*, *HLA-B40*, and *HLA-B5*, where found elevated in MS patients, while *HLA-A10* showed a protective effect against the disease. Some HLA class II antigens, specifically *HLA-DR3*,* HLA-DR4*, and* HLA-DR5*, were also found to be higher in patients (Table 6) (Al-Nashmi et al. 2018). The associations found in the study differed to those reported in Caucasians but similar to a study on patients in another gulf country, Kuwait (Al-Shammri et al. 2004).

## Other MHC associated diseases with no data available in the Arabian Gulf population

Although there are a number of studies demonstrating the involvement of MHC genes in susceptibility to autoimmune diseases for the Arabian Gulf populations, a clear picture is still far from available. There is progress in elucidating the genetic elements of major autoimmune diseases, but many have not been adequately studied in the populations of the Arabian Peninsula region. These include the deficiency in information on Hoshimoto thyroiditis, Addison’s disease, celiac disease, polymyalgia rheumaticia, autoimmune hepatitis, polyglandulat, and Behçet’s disease (Table 7). Some of diseases, such as celiac disease, are very common among the populations of the Arabian Peninsula (Saeed et al. 2017). However, due to the lack of organized national and regional epidemiological studies, the exact levels of prevalence of many of these diseases remain unknown. More effort is needed to improve our understanding of the MHC region and its association with autoimmune diseases in the populations of the Arabian Peninsula, and the surrounding Middle East region.

**Table 7:** Autoimmune diseases and HLA association that have not been studied in the population of the Arabian Peninsula. Alleles or haplotypes in brackets are protective

Disease	Ethnicity	Alleles/haplotypes	Reference
Hashimoto’s thyroiditis	Arabian	No data or research	
	Others	HLA-DR4, HLA-DR3, HLA-DR7	(Gough and Simmonds 2007)
Addison’s disease	Arabian	No data or research	
	Others	HLA-DR3	(Gough and Simmonds 2007)
Celiac disease	Arabian	No data or research	
	Others	HLA-DQ2, HLA-DQ8	(Gough and Simmonds 2007)
Polymyalgia rheumatica	Arabian	No data or research	
	Others	HLA-DR4	(Cid et al. 1988)
Autoimmune hepatitis	Arabian	No data or research	
	Others	HLA-DRB1*04:01, HLA-DRB1*03:01, HLA-DRB3*01:01	(Oliveira et al. 2011)
Polyglandular syndrome	Arabian	No data or research	
	Others	HLA-DRB1*03, HLA-DRB1*04, HLA-DQA1*03, HLA-DQB1*02	(Weinstock et al. 2011)
Behçet’s disease	Arabian	No data or research	
	Others	[ HLA-A*03], HLA-B*51	(Wallace 2014)

## Conclusion

Published manuscripts on Arabian HLA-disease associations show inconsistent results from one population to another (e.g., IDDM, RA, and MS). The significance of studies involving Arabian populations have been compromised by methodological heterogeneity, due to low sample size and participant selection, effecting the robustness of the results. The studies have also tended not to adjust for co-morbidities, and did not provide information on whether the participants were diagnosed with any other autoimmune diseases, leading to possible statistical heterogeneity and confounding results. Hence, the sample size and study design of the included manuscripts, limited meaningful interpretation of the data.

It is evident that HLA allele and MHC haplotype frequencies vary according to ethnicity and geographic origin. Variation among closely related ethnic groups can be classified by haplotype distribution, LD analysis and different allele frequencies (Hajjej et al. 2018). Manuscripts on the MHC region of the Arabian Peninsula have presented allele or haplotype associations. Yet, the majority of the reports have been based on serological methods and low resolution DNA-based HLA typing. Hence, the accuracy of HLA genotyping is particularly important to confirm disease associations.

Challenges faced by HLA-disease association studies include incomplete penetrance, interactions between genes, epigenetic factors, and environmental influences which all contribute to the clinical manifestation and phenotype severity of the disease, making it difficult to reach a conclusive genetic association (Dendrou et al. 2018). Disease association studies are negatively impacted by the rising number of HLA alleles and the ambiguity from the use of traditional methods (e.g., SSOP), compared to sequencing methods. To overcome these limitations, Next Generation Sequencing (NGS) methods may be used to provide consistent levels of resolution of HLA typing (Brown et al. 2016), and collaborative workshop activities focused on Middle Eastern populations may encourage further research in the region.

As suggested, typing based on NGS should provide the appropriate level of analytical specificity and sensitivity, reduce ambiguity, and provide more accurate allelic information for disease association studies (Profaizer et al. 2020). Publications describing DNA-based HLA typing are increasing, and with the decreasing cost of next generation sequencing technology (Klasberg et al. 2019), there is no doubt that the pool of data and our understanding of MHC disease associations will improve. NGS technology has proven its ability in identifying novel HLA alleles undetected by the clinical Sanger sequencing-based typing (SBT) (Brown et al. 2016). Particularly, NGS has successfully utilized to detect 33 novel alleles in Saudi Arabian potential bone marrow donors (Bishara et al. 2020), despite allelic definitions of HLA in public databases remain limited.

It has been well established that HLA genes are in strong long-range linkage disequilibrium leading to the existence of “conserved extended” (CEH) (Yunis et al. 2003) or “ancestral” (AH) (Degli-Esposti et al. 1992) haplotypes. These MHC haplotypes are not transmitted randomly through generations. Rather, there is transmission of specific MHC genomic subregions comprising hundreds of kilobases in length (Dawkins et al. 1999). Therefore, the susceptibility for diseases should be looked at from the perspective of MHC ancestral haplotypes and their recombinants (Dawkins and Lloyd 2019).

The MHC ancestral haplotype and the genetic architecture of the Arabian Peninsula population is not well documented, if at all. Studying Arabian MHC ancestral haplotypes, is important to provide a reference for the presence and prevalence of HLA haplotypes and alleles in the population (Kishore and Petrek 2018). This will augment research on HLA-disease susceptibility and improve histocompatibility matching for hematopoietic stem cells and bone marrow (Ameen et al. 2020) for patients of this region.

Construction of MHC ancestral haplotypes in the region could be possible through family studies as they improve the statistical power of analyses (Jahromi and Al-Ozairi 2019), and provide the opportunity to dissect the specific MHC region of interest (Dawkins et al. 1999). Finally, this review highlights the need for greater effort into characterizing the MHC region and associated diseases for the Arabian population. This is needed to assist in early diagnosis, future prevention and treatment of the disease, in addition to optimizing the matching of bone marrow donor and recipient pairs, as well as the organ distribution for transplant purposes in the region.

## Data Availability

This review discuses published data
